# The peroxins BcPex8, BcPex10, and BcPex12 are required for the development and pathogenicity of *Botrytis cinerea*

**DOI:** 10.3389/fmicb.2022.962500

**Published:** 2022-09-06

**Authors:** Ling Li, Meng-xue Yu, Jian Guo, Zhong-na Hao, Zhen Zhang, Zi-qi Lu, Jiao-yu Wang, Xue-ming Zhu, Yan-li Wang, Jie Chen, Guo-Chang Sun, Fu-cheng Lin

**Affiliations:** ^1^State Key Laboratory for Managing Biotic and Chemical Treats to the Quality and Safety of Agro-Products, Institute of Plant Protection and Microbiology, Zhejiang Academy of Agricultural Sciences, Hangzhou, China; ^2^The Key Laboratory for Quality Improvement of Agricultural Products of Zhejiang Province, College of Advanced Agricultural Sciences, Zhejiang Agriculture and Forest University, Hangzhou, China; ^3^College of Food and Health, Zhejiang Agriculture and Forest University, Hangzhou, China; ^4^College of Forestry and Biotechnology, Zhejiang Agriculture and Forest University, Hangzhou, China

**Keywords:** *Botrytis cinerea*, peroxisome, peroxins, fatty acid metabolism, pathogenicity

## Abstract

Peroxisomes have been proved playing roles in infection of several plant pathogens. Although the contribution of a portion of peroxins in pathogenicity was demonstrated, most of them are undocumented in fungi, especially, *Botrytis cinerea*. The homologs of Pex8, Pex10, and Pex12 in *B. cinerea* were functionally characterized in this work using gene disruption strategies. Compared with the wild-type strain (WT), the Δ*bcpex8*, Δ*bcpex10*, and Δ*bcpex12* mutants exhibited significant reduction in melanin production, fatty acid utilization, and decreased tolerance to high osmotic pressure and reactive oxygen species (ROS). The mycelial growth and conidiation of were significantly inhibited in Δ*bcpex8*, Δ*bcpex10*, and Δ*bcpex12* strains. The mycelial growth rates of Δ*bcpex8*, Δ*bcpex10*, and Δ*bcpex12* were reduced by 32, 35, and 34%, respectively, compared with WT and ectopic transformant (ET), and the conidiation was reduced by approximately 89, 27, and 88%, respectively. The conidial germination, germ tube elongation, and the formation of initiate infection structures (IFSs) were also reduced by the deletion of the genes. The pathogenicity was tested on the leaves of tobacco and strawberry, and fruits of tomato. On the leaves of tobacco and strawberry, the Δ*bcpex8*, Δ*bcpex10*, and Δ*bcpex12* mutants could not induce necrotic lesions, and the lesions on tomato fruits infected with the mutants were significantly reduced than those of the wide type. The results indicated that *BcPEX8*, *BcPEX10*, and *BcPEX12* are indispensable for the development and pathogenicity of *B*. *cinerea*.

## Introduction

*Botrytis cinerea*, a typical phytopathogen with a necrotrophic lifestyle infecting more than 1,000 species worldwide, has induced huge losses in agro products (Qin et al., 2010; Weiberg et al., 2013; Cui et al., 2021). Nearly 40 different strains of *B. cinerea* have been isolated and identified according to the differences in infection, pathogenicity, and biological traits (Walker et al., 2011). It was nominated for the second-rank order of fungal plant pathogen based on the scientific/economic importance (Dean et al., 2012). The pathogen infection that occurred in the field remains quiescent during the growing season and develops even at 0°C after harvesting (Romanazzi et al., 2016). The pathogen spreads among hosts by aerial mycelial growth and conidia. Effective inhibition of the mycelial growth and conidiation of *B. cineara* are the key factors to minimize gray mold development.

Peroxisomes are a type of single membrane-bound organelles that are present in almost all kinds of eukaryotes. A variety of crucial metabolic processes are commonly in the peroxisome, such as the fatty acid β-oxidation and the degradation of the reactive oxygen species (ROSs) (Reumann and Bartel, 2016; Zhang et al., 2022). The proteins involved in the peroxisome biogenies were normally assigned as peroxins, the peroxin-encoding genes were named as *PEX* genes, and up to now, there are 37 *PEX* proteins described (Kiel et al., 2006; Jansen et al., 2020). The disruption of *PEX* genes in yeasts impeded the growth on a medium that contain fatty acids as the only carbon source (Maggio and Keller, 2004). In filamentous fungi, peroxisomes also play the roles in some specific biochemical pathways, such as the biosynthesis of β-lactam antibiotics (Weber et al., 2012). Furthermore, Woronin body, a fungal-specific type of peroxisomes is required to plug the septal pores to prevent cytoplasmic leakage upon hyphal damage (Tenney et al., 2000).

A large portion of plant fungal pathogens generate conidia to reproduce and spread. Landing on the host surface, the conidia lose the nutrition resource from hyphae and have to deplete the storage component to complete the infection morphogenesis. For instance, the conidia of rice blast fungus *Magnaporthe oryzae* form appressorium, a hemispherical swollen infection structure. The appressorium is equipped high-strength cell walls and high concentration of glycerol accumulates in the interior to produce huge osmotic pressure, which pushed the penetration and infection process (Falter and Reumann, 2022). During this progress, the fatty in the conidia of *M. oryzae* is rapidly depleted to provide material and energy for the infection morphogenesis. In addition, pathogens must overcome ROSs generated in the host cells for successful invasion (Li et al., 2017). As the main organelles for fatty metabolism and ROSs degradation, peroxisomes were shown to be crucial for the pathogenicity of *M. oryzae*, as well as other fungal plant pathogens (Ramos and Naqvi, 2006; Min et al., 2012). The Δ*mgpex6* mutant of *M. oryzae* is incapable of β-oxidation of long-chain fatty acids and lack of appressorial melanin and host penetration (Ramos and Naqvi, 2006). The number of peroxisomes was found upregulated in *M. oryzae* responding to the oxidative stress generated by the host cells (Chen et al., 2017). *FgPEX5* and *FgPEX6* genes are critical to virulence and survival of *Fusarium graminearum* (Min et al., 2012). *FgPEX13*, *FgPEX14*, and *FgPEX33* play the critical roles in DON biosynthesis and virulence in *F. graminearum* (Chen et al., 2018). Our previous findings showed that quite a few *PEX* genes (*MoPEX1/5/7/11/13/14/17/19/33*) are required for the development and pathogenicity of *M. oryzae* (Wang et al., 2013, 2015, 2019; Li et al., 2014, 2017).

The conidia of *B*. *cinerea* that germinate on plant surfaces form appressorium-like structures to facilitate host penetration (Gourgues et al., 2004). Mycelia of the pathogen are also able to form highly melanized infection cushions to promote the host invasion (Marschall and Tudzynski, 2016; Cao et al., 2016; Liu et al., 2016). In addition, occasionally, host invasion by the pathogen can occur *via* germ tube apices (Van den Heuvel and Waterreus, 1983; Choquer et al., 2007). These facts reflect the infection processes and mechanism of *B. cinerea* largely differ to that of *M. oryzae*. However, to date, whether and how the peroxisome matrix protein import play the roles in the growth, development, and pathogenicity of *B. cinerea* is still not well studied. In this article, we investigated the roles of three *PEX* genes (*PEX8*, *PEX10*, and *PEX12*) of *B. cinerea* in fugal development and pathogenicity. The results certainly bring a value-add to our understanding of the regulation of *PEX8* and ring finger complex genes (*PEX10* and *PEX12*) in the growth, development, and pathogenicity of *B. cinerea*, which is vital to effective disease management.

## Materials and methods

### Fungal strains, culture conditions, and transformation

*Botrytis cinerea* wild-type (WT) B05.10 and all transformants (*BcPEX8*, *BcPEX10*, and *BcPEX12*) were cultured on a complete medium (CM) at 22°C for 3–15 days. All fungal transformants were generated by *Agrobacterium tumefaciens*-mediated transformation (*At*MT). CM plates containing 250 μg/ml hygromycin B (Roche, Mannheim, Germany) were used to screen the corresponding transformants. For dry weight measurement, mycelia of the strains were collected by culture in liquid CM with shaking.

### Sequence analysis

The homologs of Pex8, Pex10, and Pex12 in *B. cinerea* were identified by searching the NCBI database.^1^ The coding sequences of *BcPEX8*, *BcPEX10*, and *BcPEX12* were determined by amplification (with the primers set bcpex8-innerF1/bcpex8-innerR1, bcpex10-innerF1/bcpex10-innerR1, and bcpex12-innerF1/bcpex12-innerR1) from total RNAs of *B. cinerea* and sequencing the amplificons. Sequence alignments were performed using the Clustal W method and imported into the software GeneDoc2.0 for type setting and into MEGA version 7.0 to establish the phylogenetic trees.

### Gene deletion and mutant verification

Upstream fragments at least 1.5 kb equipped with *Bam* HI/*Pst*I and downstream fragments with *EcoR*I/*Xho*I for *BcPEX8*, *BcPEX10*, and *BcPEX12* were amplified, respectively, with the primer sets BCPEX8UP-P1/BCPEX8UP-B1, BCPEX10UP-P1/BCPEX10DN-B1, BCPEX8UP-P1/BCPEX8U P-B1, BCPEX8DN-E1/BCPEX8DN-X1, BCPEX10DN-E1/BC PEX10DN-X1, and BCPEX12DN-E1/BCPEX12DN-X1 and the genomic DNA of *B. cinerea* as a template. The resulting amplicons were then inserted into p1300-KO to generate the gene replacement vector Pko-BcPEX8, Pko-BcPEX10, and Pko-BcPEX12 (Supplementary Table 1).

The *PEX8*, *PEX10*, and *PEX12* mutants and ET (ectopic transformant) were generated using *Agrobacterium tumefacien*s-mediated transformation (*At*MT) (Rolland et al., 2003). The potential mutants and ET strain were first screened on CM plate with 250 μg/ml hygromycin B. Most primary transformants presumably are heterokaryons; therefore, elimination of the parental nuclei was attempted (Rolland et al., 2003). To this end, three rounds of single spore isolation and subculturing under selective pressure were performed when the transformants produced conidia.

For the verification of mutants and ET transformants, the potential strains that were resistant to hygromycin B were harvested and initially screened by genomic PCR. The Δ*bcpex8* mutants and ET transformants were checked with the primer sets bcpex8-innerF1/bcpex8-innerR1, bcpex8-outF1/Seq-BP1, bcpex8-outR1/Seq-EX1, and HPH52/HPH34. The Δ*bcpex10* mutants were checked with the primers sets bcpex10-innerF1/bcpex10-innerR1, bcpex10-outF1/Seq-BP1, bcpex10-outR1/Seq-EX1, and HPH52/HPH34. The Δ*bcpex10* mutants were checked with the primer sets bcpex12-innerF1/bcpex12-innerR1, bcpex12-outF1/Seq-BP1, bcpex12-outR1/Seq-EX1, and HPH52/HPH34. The potential deletants and ET were further confirmed by quantitative reverse transcription PCR using the primer sets PEX8RTF/PEX8RTR, PEX10RTF/PEX10RTR, and PEX12RTF/PEX12RTR (Supplementary Table 2).

### Pathogenicity tests

For pathogenicity assays, the detached leaves from tobacco and strawberry and tomato fruits were inoculated with the hypha stripped from the mycelial plugs (5 mm in diameter, 4-day-old cultures) of the strains and were then darkly incubated in a moistened box at 25°C. The symptoms of inoculated materials were recorded at 3 days.

### Phenotypic assay of the strain

Phenotypic assays for mycelial growth, conidiation, sclerotia formation, and conidial germination were performed as previously described (Hou et al., 2020).

For conidiation assay, conidia were harvested from 10-day-old of the tested strains with 2 ml ddH_2_O. Conidial concentration was estimated by counting the cells on a hemocytometer under a microscope.

For conidial germination and IFSs formation assays, 10 μl of conidia suspension (1 × 10^5^ conidia/ml) in the absence or presence of exogenous fructose (10 mM) was dropped on glass slides or onion epidermis, respectively. Then, the inoculated slides or onion epidermis were incubated in dark in a moistened box at 22°C. The number conidia germination and appressorium formation were counted under a microscope. The number of conidia germination and appressorium were counted under a microscope. At least 100 conidia were counted per replicate in each experiment; three independent experiments with triplicated per experiment were performed. The morphology of conidia and appressorium were stained with Calcofluor white (CFW) (10 μg/ml) and observed under a fluorescence microscope.

For stress adaptation assay, mycelial plugs (5 mm in diameter) taken from the periphery of a 4-day-old colony of each strain were inoculated on CM supplemented with the osmotic stress agents (NaCl and KCl, 1 M each), oxidative stress agent (H_2_O_2_, 5 mM and 7.5 mM), cell wall integrity test reagent (Congo red, 200 μg/ml and CFW 50 μg/ml), or sensitivity to fungicides (Fludioxonil,0.05 μg/ml). The inoculated CM plates were incubated at 22°C dark. At 3–4 days post-incubation, the adaptation abilities of the tested strains to the agents were observed and evaluated by their colony diameters. At least three independent experiments were performed, and in each experiment, triplicate colonies for each strain were analyzed.

For the fatty utilization assay, mycelial plugs (5 mm in diameter) taken from the periphery of a 4-day-old colony of each strain were inoculated on minimal medium (MM), which removes the glucose and supplemented with 1% (v/v) Tween80, Olive oil, 50 mM NaAC, 10 mg/ml maltase, or 10 mg/ml sucrose, respectively.

### Statistical analysis

All the quantitative data in this study were derived from at least three independent experiments with triplicate treatments. The significance between the control and the other experimental data was assessed using the Student’s *t*-test. A *p* < 0.01 was considered as significant difference.

## Results

### Identification of BcPEX8, BcPEX10, and BcPEX12 gene in Botrytis cinerea

**FIGURE 1:**
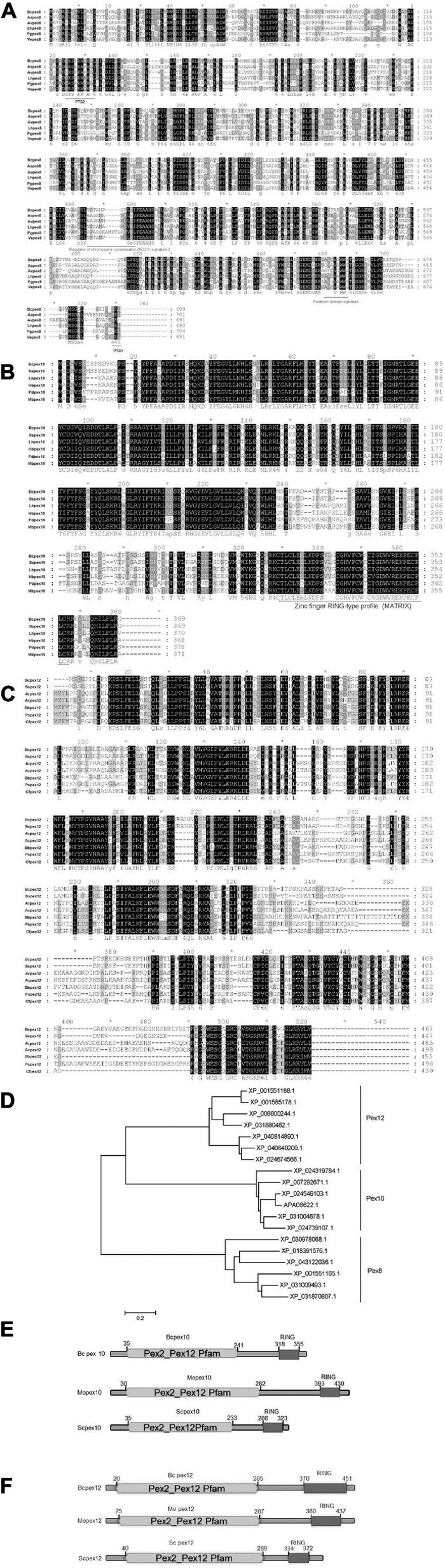
Sequence analysis of Bcpex8, Bcpex10, and Bcpex12 homologs. Protein sequences of Mopex8 **(A)**, Mopex10 **(B)**, and Mopex12 **(C)** and the homologous proteins from other related fungal species were aligned using Clustal X method. The identical amino acids are highlighted against a black background, the conserved residues on a dark gray background and the similar residues on a light gray background. “*” indicates only the positions each 10 residues. **(D)** Phylogenetic relationship of Pex8, Pex10, and Pex12 homologs calculated using the neighbor-joining method with the MEGAX program according to the alignment. **(E)** Domain architectures of *PEX10* in *B. cinerea*, *M. oryzae*, and *S. cerevisiae*. **(F)** Domain architectures of *PEX12* in *B. cinerea*, *M. oryzae*, and *S. cerevisiae*.

The potential homologous genes of *PEX8* (BCIN_16g01260), *PEX10* (BCIN_01g02750), and *PEX12* (BCIN_12g06240) in *B. cinerea* were retrieved by searching the databases (see text footnote 1) using the homologs from yeasts and related fungal species and assigned as *BcPEX8*, *BcPEX10*, and *BcPEX12*, respectively. The real coding regions of the three genes were confirmed by amplifying the cDNA fragments and were found to be identical to the hypothetical versions in the genome database. *BcPEX8* has a 2070-bp open reading frame (ORF), encoding 689 amino acid residues that show 90.42% identity to Pex8 from *Sclerotinia sclerotiorum* (XP_001585316.1) and 57% to Pex8 from *Colletotrichum tofieldiae* (KZL65398.1). Interestingly, a peroxisomal targeting signal 1 (PTS1, Ser-Lys-Leu) was present at the C terminus of Bcpex8p (Figures 1A,D), which is known to be sufficient to target proteins in peroxisome lumen in eukaryotes. Moreover, there was an internal amino acid stretch (amino acids 120–129) that resembled the proposed consensus sequence for a PTS2. The ORF of Bcpex10 is 1,110 bp long, encoding a 369-amino acid peptide with 92.41% identity to Pex10 in *Sclerotinia sclerotiorum* (XP_001595000.1) and 74.25% to Pex10 in *Lachnellula hyalina* (XP_031004878.1) (Figures 1B,D). A zinc finger RING-type profile (MATRiX) domain was found at the C terminals of the Bcpex10. The ORF of Bcpex12 is 1,404 bp long, encoding a 467-amino acid peptide with 84.14% identity to Pex12 in *Sclerotinia sclerotiorum* (XP_001585178.1) and 53.03% to Pex12 in *Beauveria bassiana* (XP_008600244.1) (Figures 1C,D). SMART analysis revealed that both Bcpex10 and Bcpex12 proteins contain PEX2_PEX12 pfam and RING finger domains (Figures 1E,F). These data suggest that Pex8, Pex10, and Pex12 are the ubiquitous peroxins in eukaryotes.

### Disruption of BcPEX8, BcPEX10, and BcPEX12

**FIGURE 2:**
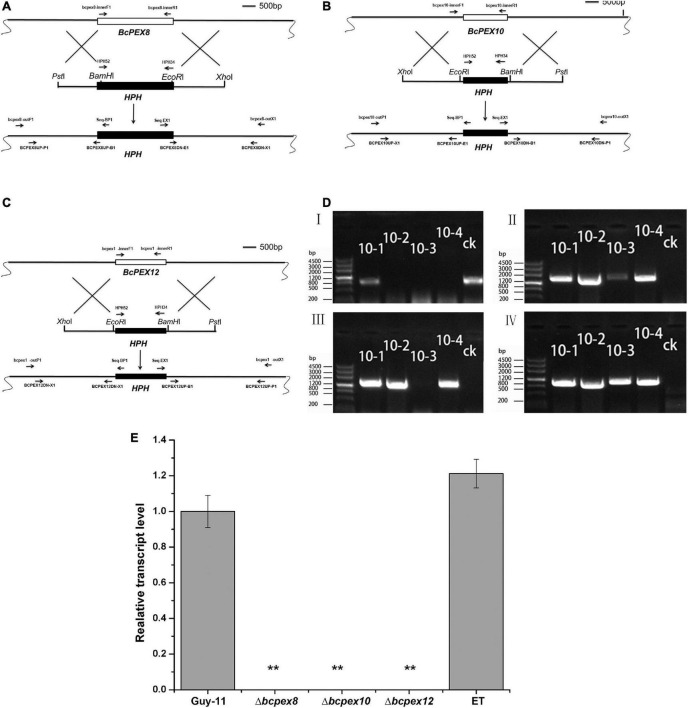
*BcPEX8*, *BcPEX10*, and *BcPEX12* genes’ deletion. **(A)** Diagram showing that the 2.07-kb *BcPEX8* coding region was replaced by the 1.36-kb *HPH* cassette. **(B)** Diagram showing that the 1.10-kb *BcPEX10* coding region was replaced by the 1.36-kb *HPH* cassette. **(C)** Diagram showing that the 1.40-kb *BcPEX12* coding region was replaced by the 1.36-kb *HPH* cassette. Scale bar, 500 bp. The locations of the primers are indicated with arrows, and primers in the same color were used as a pair. **(D)** Genomic polymerase chain reaction (PCR) was used to validate the deletion of *BcPEX10*. The upstream fragment, downstream fragment, *BcPEX10*, and *HPH* were amplified with the primer pairs Seq-BP1/bcpex10-outP1, Seq-EX1/bcpex10-outX1, bcpex10-innerF1/bcpex10-innerR1, and HPH52/HPH34, respectively. 10-1, 10-2, 10-3, and 10-4 are the possible deleted BcPEX10 mutants and ectopic transformants and ck is the wild-type strain guy-11. **(E)** Confirmation of the mutants by transcriptional analysis. *BcPEX8/10/12* transcripts were detected in the wild-type (B05.10) and ET strain using quantitative reverse transcription PCR, but completely undetectable in the mutants Δ*bcpex8*, Δ*bcpex10*, and Δ*bcpex12*. Double asterisks indicate significant differences from the transcriptional level of the wild type (*p* < 0.01).

To determine the roles of *BcPEX8*, *BcPEX10*, and *BcPEX12* in *B. cinerea*, the gene deletion mutants were generated *via* homologous recombination. The knockout vector pKO-BcPEX8, pKO-BcPEX10, and pKO-BcPEX12 were introduced into the wild-type strain B05.10 (Figures 2A–C). The hygromycin-resistant transformants of the genes were selected and checked primarily by PCR, respectively. The transformants (with *BcPEX10* as the example, and the other two genes are analogous) with the amplicon profile that bcpex10-innerF1/bcpex10-innerR1-negative, bcpex10-outF1/Seq-BP1-positive, bcpex10-outR1/Seq-EX1-positive, and HPH52/HPH34-positive were assigned as the possible deleted mutants, whereas the transformants showing a positive amplicon of bcpex10-innerF1/bcpex10-innerR1 were regarded as ectopic transformants (ET) (Figure 2D). The possible deleted mutants and ectopic transformants were selected randomly and confirmed again using qPCR. The expression of corresponding genes could not be detected in Δ*bcpex8*, Δ*bcpex10*, and Δ*bcpex12*, which indicated that the gene replacement events occurred truly in these mutants (Figure 2E). The confirmed mutants Δ*bcpex8*, Δ*bcpex10*, and Δ*bcpex12* and one of the confirmed ET strains (as the phenotypes of the ET strains for the three genes are extensively identical, that of BcPEX8 was used as a case in result figures) were used for phenotypic analysis.

### BcPEX8, BcPEX10, and BcPEX12 are required for vegetative development of Botrytis cinerea

**FIGURE 3:**
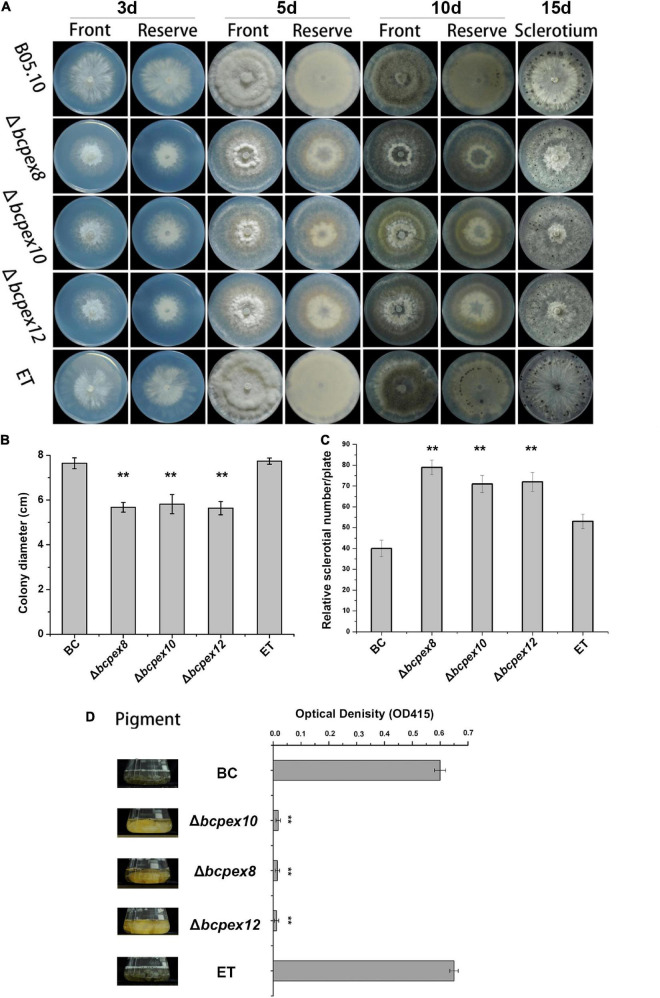
Defects in lipid utilization in Δ*bcpex8*, Δ*bcpex10*, and Δ*bcpex12*. **(A)** The mycelial growth of the Δ*bcpex8*, Δ*bcpex10*, Δ*bcpex12* mutants and WT and ET strains cultured on CM plate were measured at 3, 5, 10, and 15 days. **(B)** The colony diameters of the strains cultured on CM plate were measured at 3 days. **(C)** The sclerotium production of the strains cultured on CM plates was investigated at 15 days. **(D)** The melanin production of the strains was investigated by measuring the optical density of the sample at 415 nm (**p < 0.01).

To test whether *BcPEX8*, *BcPEX10*, and *BcPEX12* play the roles in fungal growth and development, the mycelial growth rates, melanin productions, and sclerotia production of the strains cultured on complete medium (CM) plate were measured. The data indicated that the colony diameter of the Δ*bcpex8*, Δ*bcpex10*, and Δ*bcpex12* strains was reduced significantly (Figures 3A,B). The mycelial growth of Δ*bcpex8*, Δ*bcpex10*, and Δ*bcpex12* strains was significantly inhibited, and the growth rates were reduced by 32, 35, and 34%, respectively, compared with wild-type (WT) and ectopic transformant (ET) strains at 3 days (Figure 3B). The mycelial biomass of the mutant strains was significantly reduced at 5 days (Figure 3A). The numbers of sclerotia produced by the mutant strains were more, but smaller in size, than those formed by the WT and ET strains (Figures 3A,C). The melanin productions of Δ*bcpex8*, Δ*bcpex10*, and Δ*bcpex12* mutants were significantly reduced compared with those of the WT and ET when cultured in liquid CM at 3 days (Figure 3D). These results suggest that *BcPEX8*, *BcPEX10*, and *BcPEX12* are important for *B. cinerea* mycelium growth, sclerotium, and melanin productions.

### BcPEX8, BcPEX10, and BcPEX12 are involved in asexual and IFSs development

**FIGURE 4:**
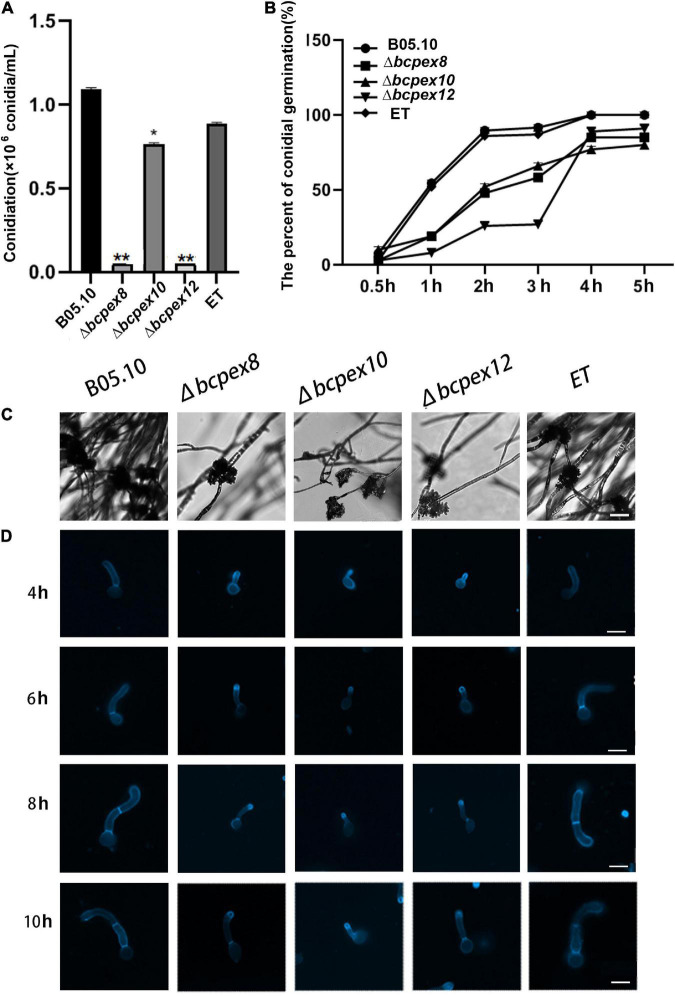
The asexual development of Δ*bcpex8*, Δ*bcpex10*, Δ*bcpex12* mutants and WT and ET strains. **(A)** Conidiation ofΔ*bcpex8*,Δ*bcpex10*, Δ*bcpex12* mutants and WT and ET strains. **(B)** The conidial germination rate ofΔ*bcpex8*,Δ*bcpex10*, Δ*bcpex12* mutants and WT and ET strains. **(C)** Observation of conidiophore cluster of Δ*bcpex8*, Δ*bcpex10*, and Δ*bcpex12* mutants and WT and ET strains. Bar = 200 μm. **(D)** Germ tube elongation of Δ*bcpex8*, Δ*bcpex10*, and Δ*bcpex12* mutants and WT and ET strains. The spores were stained with CFW. Bar = 20 μm.

Compared to the WT and ET strains, the conidiation of Δ*bcpex8*, Δ*bcpex10*, and Δ*bcpex12* was reduced by approximately 89, 27, and 88%, respectively (Figures 4A,C). The conidiophore of the mutants exhibited normal morphology, but the conidial germination of the mutants was significantly inhibited (Figure 4D). Using a fluorescence microscope, shorter germ tubes were observed in the mutants compared with WT and ET strains when incubated at 4, 6, 8, and 10 h (Figure 4B). These results show that *BcPEX8*, *BcPEX10*, and *BcPEX12* play the important roles in conidiation and germination of the fungus.

**FIGURE 5:**
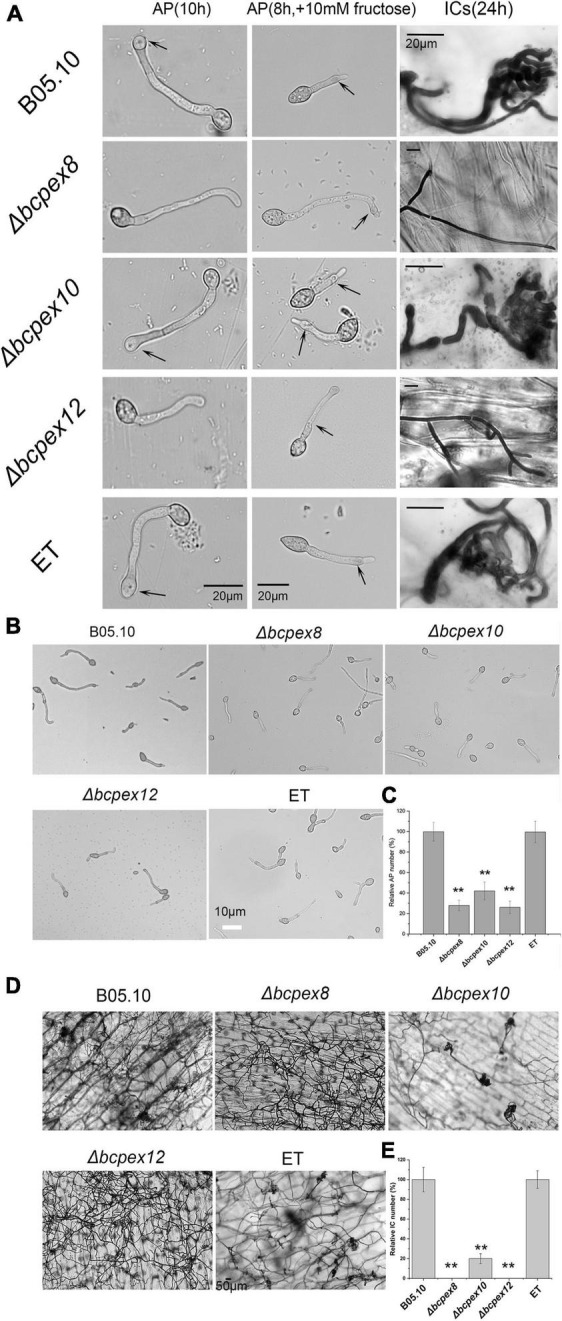
*BcPEX8*, *BcPEX10*, and *BcPEX12* are essential for the formation of IFSs. **(A)** Loss of *BcPEX8* and *BcPEX12* in of *B. cinerea* impairs appressorial or appressorium-like structure formation in the absence (leaf panel) or presence (middle panel) of 10 mM fructose at 10 and 8 hpi, respectively, and abolishes infection cushion formation (right panel) on onion epidermis at 24 hpi. **(B)** Conidia of the Δ*bcpex8*,Δ*bcpex10*, and Δ*bcpex12* mutants and WT and ET strains were performed appressorium formation assay with 10 mM fructose at 8 hpi. **(C,E)** Quantification of the relative number of formed appressoria at 10 h post-inoculation with 10 mM fructose and infection cushions at 30 hpi (h) by the indicated strains. **(D)** The indicated strains were performed infection cushion formation assay using conidia suspensions (**p < 0.01).

IFSs, including appressoria and infection cushions (ICs), play the crucial roles in host penetration in many phytopathogenic fungi. To test the effect of *BcPEX8*, *BcPEX10*, and *BcPEX12* deletion on IFSs development and melanization, we inoculated conidial suspension of WT, Δ*bcpex8*, Δ*bcpex10*, and Δ*bcpex12* mutants and ET strains on glass slides and onion epidermis to induce the formation of appressoria and ICs. All the strains formed appressoria or appressoria-like structures on glass slides at 10 h without fructose or 8 h with 10 mM fructose. The WT, Δ*bcpex10*, and ET strains formed numerous ICs at 24 h post-inoculation/incubation (hpi) (Figure 5A). However, appressoria and ICs formed by the Δ*bcpex8*, Δ*bcpex10*, and Δ*bcpex12* mutants were significantly reduced. The appressoria number of Δ*bcpex8*, Δ*bcpex10*, and Δ*bcpex12* mutants was reduced by 77.2, 58, and 74%, respectively, compared with WT and ET strains at 10 hpi (Figures 5B,C). The ICs number of Δ*bcpex8*, Δ*bcpex10*, and Δ*bcpex12* mutants was reduced by 100, 80.8, and 100%, respectively, compared with WT and ET strains at 24 hpi (Figures 5D,E). These findings demonstrated that disruption of *BcPEX8*, *BcPEX10*, and *BcPEX12* dramatically reduced appressoria and ICs formation in the mutant strains.

### Deletion of BcPEX8, BcPEX10, and BcPEX12 alters stress adaptation as well as cell wall integrity

**FIGURE 6:**
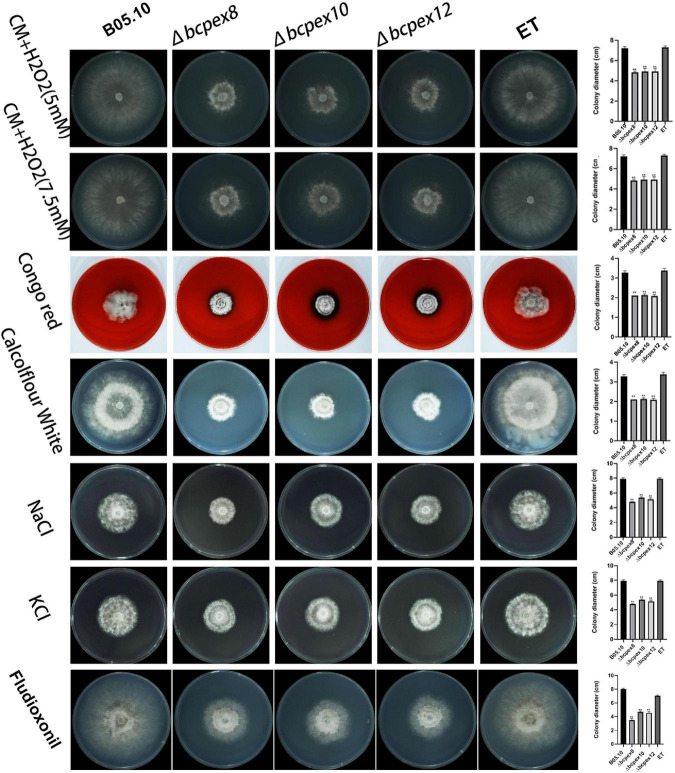
Tolerance test of Δ*bcpex8*, Δ*bcpex10*, and Δ*bcpex12* mutants and WT and ET strains to osmotic and oxidative stress, cell wall interference agent, and fludioxonil. The strains were cultured on CM plate supplemented with 5 or 7.5 mM H_2_O_2_, 200 μg/ml Congo red, 50 μg/mL Calcofluor white, 1 M sodium chloride or 1 M potassium chloride, or 0.05 μg/ml fludioxonil and were observed at 4 dpi (**p < 0.01).

To determine whether *BcPEX8, BcPEX10, and BcPEX12* mediate fungal adaption to pathogenesis-associated stress, the WT, Δ*bcpex8*, Δ*bcpex10*, Δ*bcpex12*, and ET strains were incubated on CM plates supplemented with 5 mM or 7.5 mM H_2_O_2_, 200 μg/ml Congo red (CR), 50 μg/ml CFW, 1 M NaCl/KCl, or 0.05 μg/ml fludioxonil, and colony diameters of the strains were measured at 4 days post-incubation (dpi). The growth of the Δ*bcpex8*, Δ*bcpex10*, and Δ*bcpex12* was significantly inhibited compared with the wild-type strain cultured on these media (Figure 6). The results indicated that *BcPEX8*, *BcPEX10*, and *BcPEX12* are required for the cell wall integrity, sensitivity to fungicides, and adaptation of the pathogen to oxygen stress and osmotic stress.

### BcPEX8, BcPEX10, and BcPEX12 are involved in lipid metabolism

**FIGURE 7:**
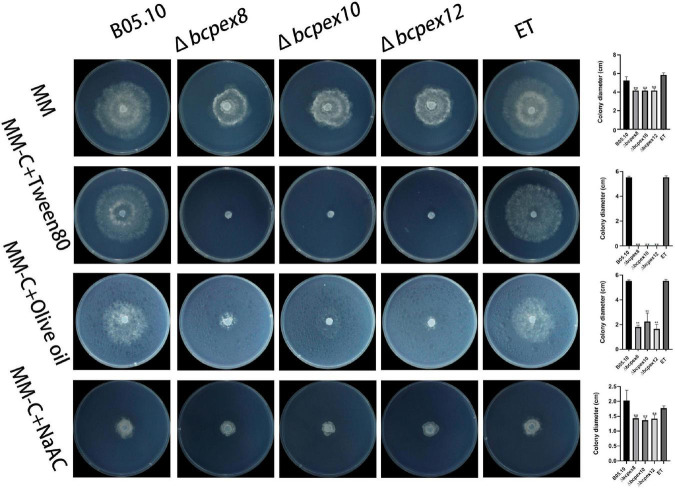
Lipid utilization assays of Δ*bcpex8*, Δ*bcpex10*, and Δ*bcpex12* mutants and WT and ET strains. The strains were cultured on MM plate supplemented with 1% tween 80, 1% olive oil, 50 mM sodium acetate, 10 g/L maltase, or 10 g/L sucrose and were observed at 3 dpi (**p < 0.01).

To assess the effects of *BcPEX8*, *BcPEX10*, and *BcPEX12* deletion on peroxisomal lipid metabolism, we investigated the lipid utilization capacity of the mutants. The development of Δ*bcpex8*, Δ*bcpex10*, and Δ*bcpex12* mutants cultured on the minimum medium (MM) complemented with Tween80, olive oil, or NaAC was significantly inhibited compared with the WT and ET strains, indicating the disruption of lipid metabolism occurred in the mutants. Moreover, Δ*bcpex8*, Δ*bcpex10*, and Δ*bcpex12* were lower efficient in the utilization of olive oil. When cultured on the MM complemented with Tween80, all the mutants failed to utilize the fatty acid (Figure 7). The results suggest that the deletion of *BcPEX8*, *BcPEX10*, and *BcPEX12* leads to the defects in lipid metabolism.

### BcPEX8, BcPEX10, and BcPEX12 are the virulence determinants of Botrytis cinerea

**FIGURE 8:**
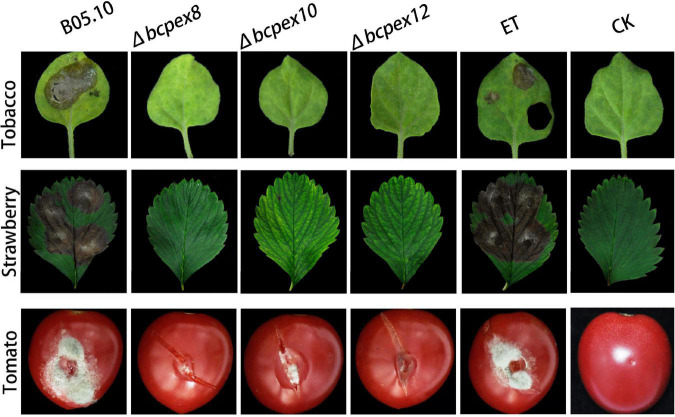
Pathogenicity of the bcpex8/10/12 deletion mutants on tobacco leaves, strawberry leaves, and tomato fruits.

To investigate whether *BcPEX8*, *BcPEX10*, and *BcPEX12* are associated with virulence, we performed pathogenicity assay with mycelia plus of the WT, Δ*bcpex8*, Δ*bcpex10*, Δ*bcpex12*, and ET strains on different plant hosts. Our findings indicated that Δ*bcpex8*, Δ*bcpex10*, and Δ*bcpex12* strains were non-pathogenic on tobacco and strawberry leaves, whereas the WT and ET strains induced severe rot symptoms on the host leaves. The Δ*bcpex8*, Δ*bcpex10*, and Δ*bcpex12* mutants could cause lesions on wounded tomato fruit in a wound-inoculation approach, but the lesions caused by the mutant strains were significantly smaller compared with those caused by the WT and ET strains (Figure 8). These findings suggest that *BcPEX8*, *BcPEX10*, and *BcPEX12* are the virulence determinants in *B. cinerea* and are required for the pathogen invasive growth upon penetration into host cells.

## Discussion

Peroxisomes are a class of monolayer organelles ubiquitously present in almost all eukaryotes, mainly involved in the β-oxidation of fatty acids and the detoxification of ROSs (Chen et al., 2018). Previous studies have shown that peroxisome and peroxisomal formation-related genes (*PEX* genes) are involved in fungal growth and development, sporulation, invasion, and parasitizing in several plant pathogenic fungi (Kimura et al., 2001; Min et al., 2012; Li et al., 2017; Wang et al., 2019). However, the roles of peroxisome and *PEX* genes in the growth, development, and pathogenicity of *B. cinerea* have not been well investigated. In this study, Pex8-, Pex10-, and Pex12-deficient mutants of *B*. *cinerea* showed similar phenotypic changes. The loss of *BcPEX8*, *BcPEX10*, or *BcPEX12* led to a plethora of defects in polar growth, melanin and sclerotium production, lipid metabolism, conidiation, and stress adaptation of the fungus.

Pex8p is an intraperoxisomal peripheral membrane protein, and it links the docking and RING finger complexes (*PEX2/10/12*) in fungi (Agne et al., 2003; Jansen et al., 2020). Less is known about *PEX8*, but has been implicated in cargo release from the PTS1 receptor *PEX5* (Ma et al., 2013). In this study, we found that the Δ*bcpex8* mutant exhibited the defects in lipid utilization and cell wall integrity, caused less lesions on the host leaves and fruits inoculated, impaired growth on agar plate, and reduced conidiation and melanin production, indicating that *BcPEX8*, as a peroxisome biogenesis factor, is involved in regulating development and pathogenicity in *B. cinerea*. It is well known that fatty acid β-oxidation is exclusively located in peroxisomes, and lipid metabolism is crucial for conidial germination and the development of infection structures in filamentous plant-pathogenic fungi (Falter and Reumann, 2022). The previous study reported that Δ*pex8* mutant of *Saccharomyces cerevisiae* failed to grow on the medium containing oleic acid (Geraghty et al., 1999), indicating that Pex8 play the roles in fatty acid utilization *S. cerevisiae*. The results in this study confirmed the association between *BcPEX8* and lipid metabolism in *B. cinerea*. Plant-pathogenic fungi use mycotoxins to alter plant metabolism for their advantage in invasion (Pusztahelyi et al., 2015). Peroxisomes are essential for mycotoxin biosynthesis in several fungal species (Falter and Reumann, 2022). Although the role of *PEX8* gene in toxin biosynthesis has not been well elucidated, a recent study reported that *FvPEX8* was a key component in *F. verticillioides* docking module affected peroxisome function and fumonisin biosynthesis (Yu et al., 2021). It will be an interesting and our undergoing topic whether the involvement of *BcPEX8* in pathogenicity is associated with mycotoxin biosynthesis in *B. cinerea*.

Pex10p and Pex12p are RING finger peroxins that have ubiquitin (E3) ligase activity and are involved in *PEX5* recycling in filamentous plant-pathogenic fungi (Falter and Reumann, 2022). To date, there are only a few reports regarding phenotypic analysis of *PEX10* and *PEX12*, and their roles in phytopathogenic fungi remain to be clearly established. Previous studies demonstrated that in the Δ*pex10* and Δ*pex12* mutants of *F. graminearum*, the conidia and hyphae were prone to be broken, the cell walls were more sensitive to the cell wall-perturbing agents, and the mycelial growth, conidiation, and lipid metabolism are impaired, compared with the wild type. *F. graminearum* Δ*pex10* and Δ*pex12* mutants also showed downregulation of selected *TRI* genes and reduced deoxynivalenol production compared with the wild type (Zhang et al., 2019; Wang et al., 2020). However, less is known about the roles of *PEX10* and *PEX12* in fungal development and pathogenicity in *B. cinerea*. Our results reveal that both *BcPEX10* and *BcPEX12* are involved in hyphal growth, asexual reproduction, fatty acid utilization, maintenance of cell wall integrity, and pathogenicity in *B. cinerea*. Comparison of the findings with the documented studies confirmed that *PEX8*, *PEX10*, and *PEX12* play the roles in the development and pathogenicity in both *B. cinerea* and *F. graminearum.* We demonstrated previously that *MoPEX1*, *MoPEX11A*, *MoPEX13*, *MoPEX14*, *MoPEX14/17*, and *MoPEX19* were required for development and pathogenicity in *M. oryzae* (Li et al., 2014, 2017; Wang et al., 2015, 2019). The collected evidence provides further support for the hypothesis that peroxisomes play numerous essential roles in fungal development and pathogenicity, and *PEX* gene has now emerged as an important virulence factor bearing the possibility to block multiple biogenesis pathways of different virulence factors simultaneously.

In the ongoing attempts to develop novel control agents against plant fungal pathogens, some peroxisomal enzymes essential for virulence are regarded as elegant targets, which implied that the peroxisome is a potential organelle used as a target. For lowering the risk of rapid evolution of fungicidal resistance in fungi, multiple gene targets were deemed to be preferred to single gene targets. Thus, more *PEX* genes in plant-pathogenic fungi are required to be further characterized. In addition, recent studies have shown that RNAs can move from a host to the interacting pathogen to inhibit infection (Cai et al., 2019). The exogenous small interfering RNAs against the *PEX* genes of plant-pathogenic fungi also have the potentiality as environmentally friendly RNA fungicides for crop protection.

In summary, the Pex8, Pex10, and Pex12 play the crucial roles in the development and pathogenicity in plant pathogenic fungus, *B. cinerea*.

## Data availability statement

The original contributions presented in this study are included in the article/Supplementary material, further inquiries can be directed to the corresponding author/s.

## Author contributions

J-YW: conceptualization. LL, ZZ, and JG: data curation. M-XY, Z-QL, and X-MZ: formal analysis. LL: funding acquisition. J-YW and G-CS: investigation. M-XY, Z-QL, ZZ, and J-YW: methodology. Y-LW and G-CS: project administration. LL, X-MZ, and ZZ: software. J-YW, G-CS, and F-CL: supervision. Z-QL and LL: validation. All authors contributed to the article and approved the submitted version.

## Footnotes

1
https://www.ncbi.nlm.nih.gov/
