# Supplementary material for: The peroxins BcPex8, BcPex10, and BcPex12 are required for the development and pathogenicity of Botrytis cinerea
Source: Front Microbiol. 2022 Sep 6;13:962500. doi: 10.3389/fmicb.2022.962500 (PMC9488000; doi:10.3389/fmicb.2022.962500)
Supplement: Supplementary file 1 [file Data_Sheet_1.docx]

Table 1 Primers used in this study

| **Name** | **Sequence（5’- 3’）** |
| --- | --- |
| BCPEX8UP-P1 | GCCAAGCTTGCATGCCTGCAG |
| BCPEX8UP-B1 | ATGTGTTGACCTCCAGGATCC |
| BCPEX8DN-E1 | CCGGGTACCGAGCTCGAATTC |
| BCPEX8DN-X1 | ATTATTATGGAGAAACTCGAG |
| BCPEX10UP-E1 | CCGGGTACCGAGCTCGAATTC |
| BCPEX10UP-X1 | ATTATTATGGAGAAACTCGAG |
| BCPEX10DN-P1 | GCCAAGCTTGCATGCCTGCAG |
| BCPEX10DN-B1 | ATGTGTTGACCTCCAGGATCC |
| BCPEX12UP-P1 | GCCAAGCTTGCATGCCTGCAG |
| BCPEX12UP-B1 | ATGTGTTGACCTCCAGGATCC |
| BCPEX12DN-E1 | CCGGGTACCGAGCTCGAATTC |
| BCPEX12DN-X1 | ATTATTATGGAGAAACTCGAG |

Table 2 Primer sequences were used to validate the disruption of *BcPEX8*, *BcPEX10* and *BcPEX12* in this study

| **Name** | **Sequence（5’- 3’）** |
| --- | --- |
| bcpex8-innerF1 | GGAGGGAGGCATGGGCTCTCTAGC |
| bcpex8-innerR1 | CTGACTATCTTGCTGGCTGGCGAT |
| bcpex8-outF1 | GTCTTCTATCAGATGTCATTGGGC |
| bcpex8-outR1 | GGGAATGAGAGCCGTGGCAATACC |
| bcpex10-innerF1 | CCTATCAGTACCCATTCGCAGCAG |
| bcpex10-innerR1 | ACATGTCCACATCCCAACACACTC |
| bcpex10-outF1 | GATGTGCTAATGCGCGATTGAGAC |
| bcpex10-outR1 | TCATCCACTCCCGGGCCACCTAGC |
| bcpex12-innerF1 | CGAACTCCTGTCCTCAACTCAACT |
| bcpex12-innerR1 | CTCGCCGCTCGCTACAACCTCTGC |
| bcpex12-outF1 | TCTGTTCATGCCAATTCGGGTCCG |
| bcpex12-outR1 | TGATTGCAGATTACTCCTCAGATA |
| HPH52 | AGCTGCGCCGATGGTTTCTACAA |
| HPH34 | GCGCGTCTGCTGCTCCATACAA |
| Seq-BP1 | TGCTCACCGCCTGGACGACTAAAC |
| Seq-EX1 | CGATAGTGGAAACCGACGCCCC |
| PEX8RTF | CTACCTCTCTACTTACCACTCTTA |
| PEX8RTR | GTTTTTGTGCACTGTAATGGCAGC |
| PEX10RTF | TCGGATGTCAAGGGTCCATCCTAT |
| PEX10RTF | GTATACGTGTGAAGAAATCTTGCT |
| PEX12RTF | TCTGGCTGATAGGTACCCGCATAC |
| PEX12RTF | ATCCAGCCCCTAATCTTCCCATGG |
